# Estimating Trait Heritability in Highly Fecund Species

**DOI:** 10.1534/g3.115.020701

**Published:** 2015-10-04

**Authors:** Sarah W. Davies, Samuel V. Scarpino, Thanapat Pongwarin, James Scott, Mikhail V. Matz

**Affiliations:** * Department of Integrative Biology, The University of Texas at Austin, Texas 78712; ‡ Department of Statistics and Data Sciences, The University of Texas at Austin, Texas 78712; § Department of Information, Risk, and Operations Management, The University of Texas at Austin, Texas 78712; † Santa Fe Institute, Santa Fe, New Mexico 87501

**Keywords:** heritability, nonmodel organisms, common garden, binary variable traits, coral settlement

## Abstract

Increasingly, researchers are interested in estimating the heritability of traits for nonmodel organisms. However, estimating the heritability of these traits presents both experimental and statistical challenges, which typically arise from logistical difficulties associated with rearing large numbers of families independently in the field, a lack of known pedigree, the need to account for group or batch effects, etc. Here we develop both an empirical and computational methodology for estimating the narrow-sense heritability of traits for highly fecund species. Our experimental approach controls for undesirable culturing effects while minimizing culture numbers, increasing feasibility in the field. Our statistical approach accounts for known issues with model-selection by using a permutation test to calculate significance values and includes both fitting and power calculation methods. We further demonstrate that even with moderately high sample-sizes, the p-values derived from asymptotic properties of the likelihood ratio test are overly conservative, thus reducing statistical power. We illustrate our methodology by estimating the narrow-sense heritability for larval settlement, a key life-history trait, in the reef-building coral *Orbicella faveolata*. The experimental, statistical, and computational methods, along with all of the data from this study, are available in the R package multiDimBio.

Organisms with high fecundity, small propagule size, and limited parental investment, also referred to as r-selected species, often exhibit higher levels of nucleotide diversity and/or standing genetic variation compared with k-selected species (Romiguier *et al.* 2014). Many marine species, including fish and invertebrates, exhibit these r-selected life history characteristics (Doherty and Fowler 1994) and indeed have been shown to exhibit high levels of genetic diversity (Bay *et al.* 2004; Davies *et al.* 2015). However, this high genetic diversity does little to predict how a population will respond to environmental perturbations, such as those caused by climate change. Instead, the key question is not how much variation is present, but what is the heritability of the traits under selection after the perturbation. Quantifying narrow-sense heritability, the proportion of phenotypic variance attributable to additive genetic effects (Lynch and Walsh 1998), for nonmodel organisms presents both experimental and statistical challenges. Most experiments aiming to quantify narrow-sense heritability involve multigenerational breeding programs and large numbers of crosses with many culture replicates to account for “jar effects,” both of which are rarely feasible in nonmodel species.

Here we present a quantitative genetic methodology for estimating the narrow-sense heritability of traits in highly fecund species. The method does not require the onerous sampling schemes usually required for these types of experiments. Instead, our approach leverages high fecundity by completing independent fertilizations with large quantities of eggs equally divided among sires to account for sperm competition (Figure 1). These cultures are then mixed into a single bulk culture (common garden) and divided into three replicate tanks per dam. Bulk larvae are then sorted according to the trait of interest, which in this study is a binary trait (whether or not the larvae underwent metamorphosis in response to settlement cue). Single larvae that “succeeded” and “failed” are then individually genotyped and their sire assignments are compared with the predicted distribution of sire assignments in the original design. This experimental design allows for all sires to be cultured in “common garden” conditions, which greatly reduces the number of cultures compared with a standard approach, where each family would be cultured individually, resulting in a culture number of 3× the number of sires. The narrow-sense heritability of these data can be estimated using a generalized linear mixed model with a binomial error distribution. However, as we discuss herein, appropriately determining statistical significance is nontrivial. This method of quantifying heritability of binary traits is broadly applicable to many traits of interest including—but not limited to—stress tolerance, dispersal potential, and disease susceptibility. Furthermore, the framework we have developed—including the statistical methods—can be readily adapted to traits with different distributions, *e.g.*, normally distributed phenotypes.

**Figure 1:**
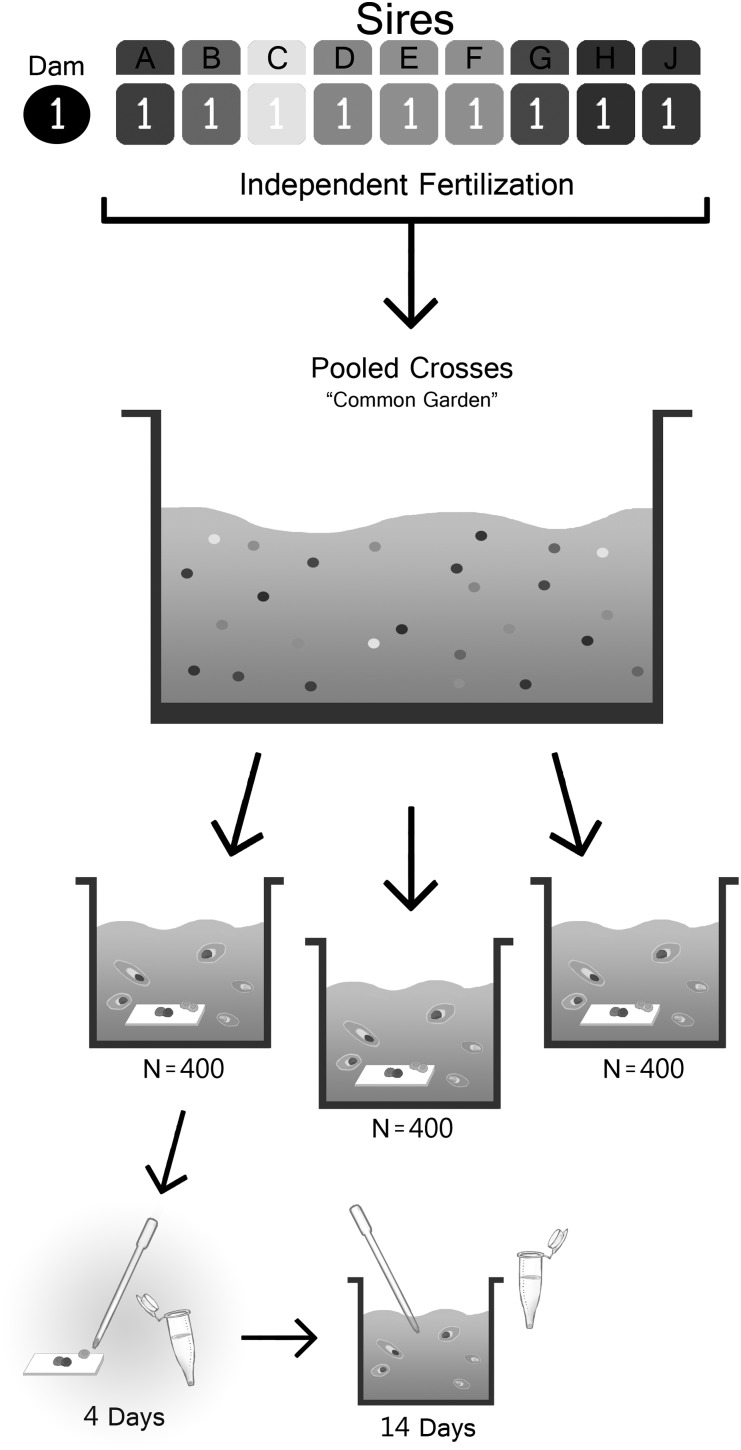
Diagram representing the design of the common garden experiment. First, independent fertilizations are completed for each sire and dam (in this case only one dam and nine sires are used). Second, equal quantities of fertilized embryos are pooled into one single common garden tank. This common garden is the split into three replicate tanks (N = 400 larvae per tank). Settlement slides are added to each experimental tank and after 4 days the settled larvae are collected and individually preserved. Larvae were then left for an additional 10 days and settled larvae were removed every few days. N = 50 larvae that remained swimming after 14 days were collected and individually preserved for genotyping, to compare their parentage to the parentage of the early-settling larvae.

To demonstrate this methodology, we estimated the heritability of dispersal potential in reef-building coral larvae. The majority of corals—like many other marine invertebrates—release gametes into the water annually. These gametes develop into planktonic larvae that are dispersed by ocean currents, representing each coral’s only dispersal opportunity (Baird *et al.* 2009). The now pelagic larvae can travel great distances before settling on a reef, but once the larva settles, it will remain there for the duration of its life. Therefore, selection for dispersal potential is limited to optimizing larval traits, which can be investigated through classical quantitative genetics, *e.g.*, Meyer *et al.* (2009). Specifically, we determined how much variation in the early larval responsiveness to settlement cue depends on the genetic background of larvae. The experiments were performed on larvae of the hermaphroditic mountainous star coral, *Orbicella faveolata*, which is an important but endangered Caribbean reef-building coral. To analyze these data, and estimate the narrow-sense heritability of this binary trait, we developed a Monte Carlo method for performing model selection and calculating statistical power with generalized linear mixed models. The code and data are available in the R package multiDimBio (Scarpino *et al.* 2014).

## Materials and Methods

### Experimental framework

Our experimental framework, which is summarized in Figure 1, proceeds in four steps. First, we perform crosses between the desired number of parents. Second, all offspring from a single dam are reared in the same environment (“common garden”). Third, offspring are phenotyped for the trait of interest and genotyped to determine paternity. Fourth, these data are analyzed by the use of random-effects models and a permutation test to determine statistical significance. What follows is a detailed description of how to estimate the narrow-sense heritability of coral settlement using this framework.

### Application of the experimental framework to coral settlement

#### Crossing design and larval rearing:

One day before the annual coral spawn on August 7, 2012, 10 independent *O. faveolata* colony fragments (10 cm × 10 cm) were collected from the East Flower Garden Banks National Marine Sanctuary, Gulf of Mexico. Colonies were maintained in flow through conditions aboard the vessel and were shaded from direct sunlight. Colonies were at least 10 m apart to avoid sampling clones, as clones within reefs have been detected in this genus (Severance and Karl 2006; Baums *et al.*, 2010). However, intracolony variation (chimerism) in scleractinian corals is very rare (Puill-Stephan *et al.*, 2009), so each sire was assumed to only produce sperm of a single genotype. Before spawning, at 20:00 Central Daylight Time (CDT) on August 8, 2012, colonies were isolated in individual bins filled with 1 μL of filtered seawater and were shaded completely. Nine colonies spawned at approximately 23:30 CDT. From these spawning colonies, we collected gamete bundles and separated eggs and sperm with nylon mesh. Each colony was used as an independent sire, with no additional sperm/sires included in this study. Samples from each sire were preserved in ethanol for genotyping.

Divers collected gamete bundles directly from three colonies during spawning and eggs were separated to serve as maternal material (N = 3 dams). Eggs were divided equally among fertilization bins (N = 9 per dam) and sperm from each sire was added at 02:00 CDT on August 9, 2012, for a total of 27 fertilization bins. Control self-cross trials verified that self-fertilization was not detectable in our samples. After fertilization, at 08:00 CDT, excess sperm was removed by rinsing with nylon mesh, and embryos for each dam across all sires were pooled in one common culture. Densities were determined and larvae were stocked into three replicate culture vessels at one larva per 2 mL for a total of nine culture containers (N = 3 per dam). Larvae were transferred to the University of Texas at Austin on August 10, 2012. After spawning, colony fragments were returned to the reef. All work was completed under the Flower garden Banks National Marine Sanctuary permit #FGBNMS-2012-002.

#### Common garden settlement assay:

On August 14, 2012, 6-d-old, precompetent larvae from the three replicate bins for a single dam were divided across three settlement assays. Four hundred healthy larvae per culture replicate were added to a sterile 800-mL container with five conditioned glass slides and finely ground, locally collected crustose coralline algae, a known settlement inducer for this coral genus (Davies *et al.* 2014). Cultures were maintained for 3 d, after which each slide was removed and recruits were individually preserved in 96% ethanol, representing larvae exhibiting “early” responsiveness to settlement cue. Culture water was changed, new slides were added with additional crustose coralline algae, and larvae were maintained until they reached 14 d of age. All settled larvae on slides were discarded, and 50 larvae per culture were individually preserved in 96% ethanol. Larvae from the other two dams were not used in these assays due to high culture mortality.

#### Larval DNA extraction:

Larval DNA extraction followed a standard phenol-chloroform iso-amyl alcohol extraction protocol, see Davies *et al.* (2013), with modifications to accommodate for the single larva instead of bulk adult tissue.

#### Parental genotyping:

Sire genotyping was completed with the use of nine loci from Davies *et al.* (2014) and four loci from Severance *et al.* (2004) following published protocols. Amplicons were resolved on agarose gel to verify amplification and molecular weights were analyzed using the ABI 3130XL capillary sequencer. GeneMarker V2.4.0 (Soft Genetics) assessed genotypes and loci representing the highest allelic diversities among the sires were chosen as larval parentage markers. To ensure that each sire was a unique multilocus genotype (MLG) and that the relatedness between sires was negligible, we compared the allelic composition of each sire across six microsatellite loci (MLG) and calculated the Probability of Identity at each locus in GENALEX v6.5 from Peakall and Smouse (2006).

#### Larval parentage:

To compensate for the low larval DNA concentrations, 3 μL of each single extracted larva (unknown concentration) was amplified in a multiplex reaction with six loci from Davies *et al.* (2013) with the following modifications: 1μM of each fluorescent primer pair (N = 6) and 20-μL reaction volumes (Table 1). Alleles were called in GeneMarker V2.4.0 and offspring parentage was assigned based on presence/absence of sire alleles. Data were formatted into a dataframe consisting of the number of early settlers and swimming larvae that were assigned to each sire (A-J) from each of three replicate bins (1−3).

**Table 1:** Summary of the six microsatellite loci from Davies *et al.* (2013) used in paternity assignment

Locus	Observed, bp	*N_a_*	Fluorescence
M_fav4	375-391	5	FAM
maMS2-5	280-328	20	FAM
maMS8	197-203	3	FAM
M_fav6	387-429	11	HEX
M_fav7	453-498	9	HEX
maMS2-8	187-205	10	NED

N_a_ is the number of alleles. FAM, HEX, and NED are fluorescent dyes used for labeling the markers so that they can be multiplexed.

### Statistical methods

#### Estimating narrow-sense heritability from binary data:

In principle, estimating narrow-sense heritability for a binomially distributed trait, such as coral settlement, is straightforward, see Gilmour *et al.* (1985); Foulley *et al.* (1987); Vazquez *et al.* (2009); Biscarini *et al.* (2014, 2015). The desired quantity is the among-sire variance, denoted as *τ*^2^, which can be estimated with a generalized linear mixed model with a binomial error distribution. Although this a departure from the standard threshold approach for estimating the heritability of binomial traits, it is now fairly common in the quantitative genetics literature, see Foulley *et al.* (1987) and Vazquez *et al.* (2009).

Suppose we have binary observations $y_{ij}\in \left\{0,1\right\}$ where *i* index units (sires) and *j* indexes observations within units. The model is simple Bernoulli sampling, parameterized by log odds:

$$P\left(y_{ij}=1\right)=\frac{1}{1+\text{exp}\left(-\psi_{ij}\right)}.$$ (1)

We will assume that the log odds have a sire-level random effect:

$$\psi_{ij}=\text{α}+{\text{β}}_{i}\;,\;{\text{β}}_{i}∼N\left(0,\tau^{2}\right)\;.$$

Thus we have a simple binary logit model with a single random effect. A standard result on logit models is that we can represent the outcomes *y_ij_* as thresholded versions of a latent continuous quantity *z_ij_* (Holmes *et al.* 2006):

$$\begin{array}{l} y_{ij}=\{\begin{array}{cc} 1 & \text{if }z_{ij}\geq 0,\\ 0 & \text{if }z_{ij}<0. \end{array}\\ z_{ij}=\text{α}+{\text{β}}_{i}+{\text{ε}}_{ij}\;, \end{array}$$

where *ε_ij_* follows a standard logistic distribution. Note this nonstandard form of latent-threshold model, wherein the errors *ε_ij_* are logistic rather than normally distributed. Upon integrating out the *z_ij_* values (which are often referred to as latent or data-augmentation variables), we recover exactly the logistic regression model of Equation (1) with a sire-level random effect.

In light of this, we can interpret narrow-sense heritability in terms of the ratio of predictable to total variation in our logistic random-effects model. This is often referred to as the Bayesian *R*^2^, by analogy with the classical coefficient of determination in a regression model:

$$R^{2}=\frac{\text{var}\left({\text{β}}_{i}\right)}{\text{var}\left(z_{ij}\right)}=\frac{\text{var}\left({\text{β}}_{i}\right)}{\text{var}\left({\text{β}}_{i}\right)+\text{var}\left({\text{ε}}_{ij}\right)}=\frac{\tau^{2}}{\tau^{2}+\pi^{2}/3}\;,$$

exploiting the facts that the *β_i_* and *ε_ij_* are independent and that the variance of the standard logistic distribution is π^2^/3. The aforementioned equation for the Bayesian *R*^2^ is the narrow-sense heritability for the animal model. Therefore, the among-sire variance can be transformed into an approximation of narrow-sense heritability under the sire model by multiplying the Bayesian *R*^2^ by four, see Foulley *et al.* (1987) and Vazquez *et al.* (2009) for a more detailed derivation and Lynch and Walsh (1998) for a discussion of the assumptions this approximation relies on.

However, under this model, determining whether statistical support exists for an among-sire variance greater than zero remains a challenge. Traditionally, an approach to the problem would be to fit two models, one where*τ*^2^, the among-sire variance, is a free parameter and one where it is constrained to zero. These models can then be compared, and model selection performed, with a likelihood ratio test, or in this case the difference in each model’s deviance, which is equivalent to a likelihood ratio test for nested models. Although, critically, this is a special kind of likelihood ratio test because the null hypothesis resides on the edge of the parameter space. The large sample reference distribution for this type of test is usually considered to be a 50% mixture of a point of mass at zero and a χ^2^ (1) (Self and Liang 1987). However there is still substantial debate in the literature about what mixture should be used—*e.g.*, Crainiceanu *et al.* (2003)—and it is not clear whether any of these mixtures are valid null distributions for finite sample sizes.

Instead, our approach is to construct a permutation-based method for calculating a p value for the likelihood ratio test and performing model selection. This test is simple to implement, because it only involves randomly shuffling the identity of each offspring’s sire a large number of times (say, 500) and refitting the random-effects model to each shuffled data set. This avoids making assumptions about the asymptotic distribution of the test statistic that may fail to hold for finite sample sizes.

#### Monte Carlo simulation for the likelihood ratio test:

Our simulations assume a fixed probability of settlement, *p_setttle_*, to be equal across all sires, in this case *p_setttle_* = 0.285 (the global mean), and simulate 1000 data sets where the number of offspring for each sire in each of three bins is drawn from a negative binomial distribution with μ = 4.63 and size = ${\text{μ}}^{2}/\left(\sqrt{12.63}-\text{μ}\right)$, again these are the empirically observed values across sires. The resulting 1000 data sets have the same structure as the observed data, but the only among sire variability comes from sampling, the true *τ*^2^ = 0. For each simulated data set, we calculated the likelihood-ratio test statistic. This provides a Monte Carlo approximation to the true sampling distribution of the test statistic under the null.

#### Power analysis:

With any novel experimental design, it is desirable to construct a method for estimating its statistical power. Using the Monte Carlo approach designed to calculate p-values for likelihood ratio tests, we can simulate data sets with an arbitrary number of sires, number and variance in offspring, among-sires variance, and number of bins. By repeatedly simulating data sets with fixed combinations of these parameters, the statistical power is simply the fraction of times we correctly reject the null hypothesis. Similarly, the false-positive rate is the fraction of times we falsely reject the null hypothesis.

### Data availability

All code and data developed for this study are available in the R package multiDimBio (Scarpino *et al.* 2014). The statistical models were fit using the R packages stats in R version 3.2.1 (R Core Team 2015) and lme4 version 1.1-8 (Bates *et al.* 2015).

## Results

### Sire independence

Each sire was determined to be a unique MLG across the six microsatellite loci indicating that no clones were collected (Table 2). To ensure that each sire could be considered independent, we calculated the Probability of Identity at each locus and found that these probabilities ranged from 3.2E-01 for a single locus down to 2.0E-06 when all six loci are considered and therefore each sire was considered independent.

**Table 2:** Summary of paternity assignment results

Sire	Locus 1 MaMS8	MaMS8.1	Locus 2 Sev5	Sev5.1	Locus 3 Mfav4	Mfav4.1	Locus 4 Mfav6	Mfav6.1	Locus 5 Mfav7	Mfav7.1	Locus 6 Sev8	Sev8.1
A	200	200	280	322	379	379	391	391	453	465	190	196
B	200	203	292	322	379	379	389	391	471	486	187	190
C	200	200	283	313	375	375	419	429	453	471	190	193
D	197	200	301	322	375	379	423	423	465	486	190	196
E	200	200	283	316	375	391	389	389	453	474	190	193
F	197	197	307	313	375	375	391	391	462	471	190	202
G	197	200	301	328	379	379	391	391	474	474	193	205
H	197	200	280	307	383	383	389	389	453	453	190	193
J	197	200	280	313	379	379	389	389	477	498	193	193

Values are the microsatellite lengths for each of six loci from Davies *et al.* (2014)

### Parentage

**Figure 2:**
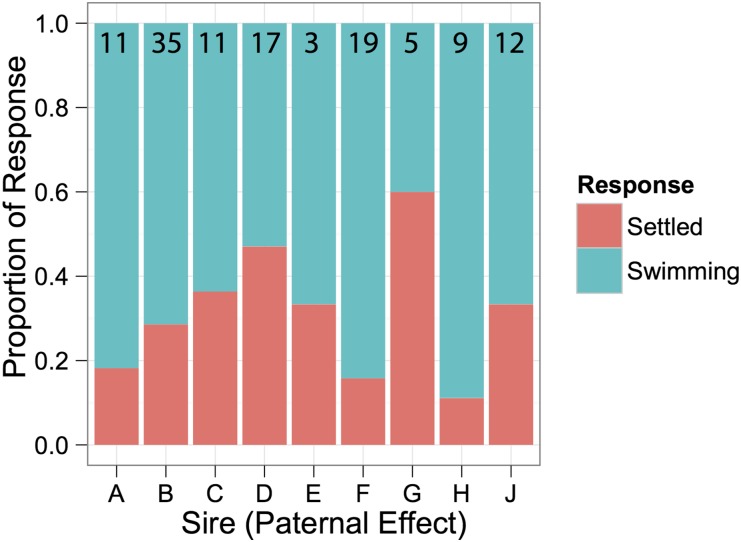
Proportion of settled (successes) and swimming (failures) larvae belonging to each sire. The total number of genotyped larvae assigning to each sire is indicated at the top of each bar.

Larvae that amplified at >2 loci were considered successful amplifications. A total number of 55 recruits (binary successes) were collected and of these 47 were amplified and 37 were assigned parentage. A total number of 129 swimming larvae (binary failures) were extracted and of these 112 amplified successfully and 81 were assigned parentage (Figure 2).

### Monte Carlo simulation for the likelihood ratio test

**Figure 3:**
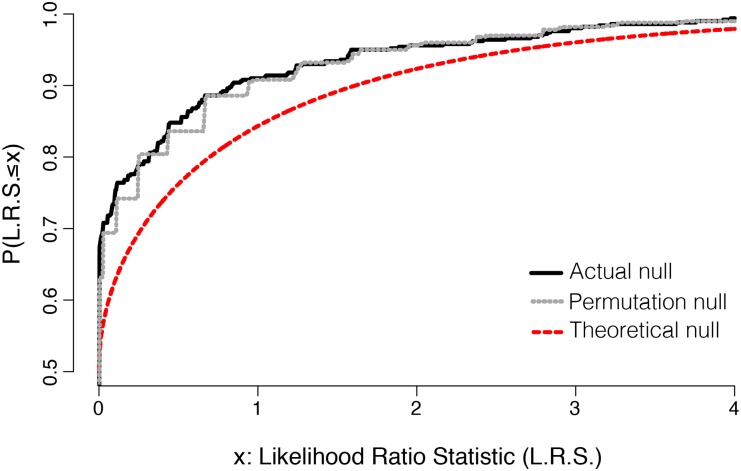
The cumulative distribution function for the actual (black solid), permutation (gray dashed), and theoretical (red dashed) nulls are compared. The permutation null is a closer match to the actual null and is less conservative than the asymptotic approximation. This suggests that asymptotic approximation to the true null distribution is inappropriate for our data set.

To test whether the procedure proposed in this study provided any benefits over the traditional approach to performing a likelihood ratio test, we first simulated the true sampling distribution of the likelihood ratio statistic under the null hypothesis. This was accomplished by repeatedly simulating data from a model where the true among-sire variance (*τ*^2^) was zero. The cumulative distribution function of this random variable is shown as a black curve (actual null) in Figure 3. We then calculated two approximations to this sampling distribution; these cumulative distribution functions are also plotted in Figure 3. First, the red curve (theoretical null) shows a mixture distribution of a point mass at 0 (with probability 0.5) and χ^2^ (1) random variable (with probability 0.5). This is the asymptotic approximation to the true null used in the traditional likelihood-ratio test of a variance component in a mixed-effects model. Second, the dotted gray curve (permutation null) shows the estimated null distribution obtained by running the permutation test on a single simulated data set. The permutation null is clearly a better approximation to the actual null than is the theoretical null, whose distribution is shifted to the right. This fact suggests that—at least for data sets similar to ours—the asymptotic approximation is too conservative, and will therefore lead to reduced power at a specified false-positive rate.

### Statistics

**Figure 4:**
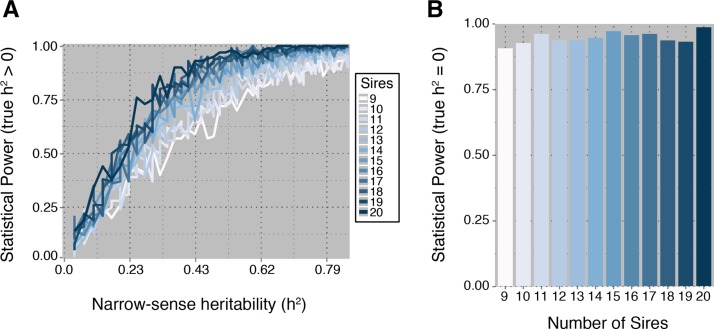
Power analysis for a varying number of sires. The offspring number was fixed, at μ = 4.63 and size = $\mu^{2}/\left(\sqrt{12.63}-\mu \right)$ respectively, and the number of sires was varied between 9 and 20. In (A), the power to reject the null hypothesis of *h*^2^ = 0 is plotted as a function of narrow-sense heritability (*h*^2^), where the true value of *h*^2^ > 0. (B) The power to fail-to-reject the null hypothesis when the true value of *h*^2^ was equal to zero is plotted for varying numbers of sires.

Using the described experimental design and statistical methods, we were unable to detect a significant random effect of sire, although there was a trend in overall variation in early settlement among sires (Figure 2). However, by bootstrapping the data, we were able to obtain an estimated *τ*^2^ of approximately 0.176 (0.42 standard deviation), corresponding to a narrow-sense heritability of around 0.2 (95% confidence interval 0.0−1.0). Considering the number of sires used and offspring sampled in our study, the true narrow-sense heritability would have to be well above 0.6 to achieve 80% power (Figure 4A). Nevertheless, this experimental set up should be sufficiently powered to correctly fail to reject the null hypothesis if in fact the true among sire variance was zero (Figure 4B).

### Power analysis

**Figure 5:**
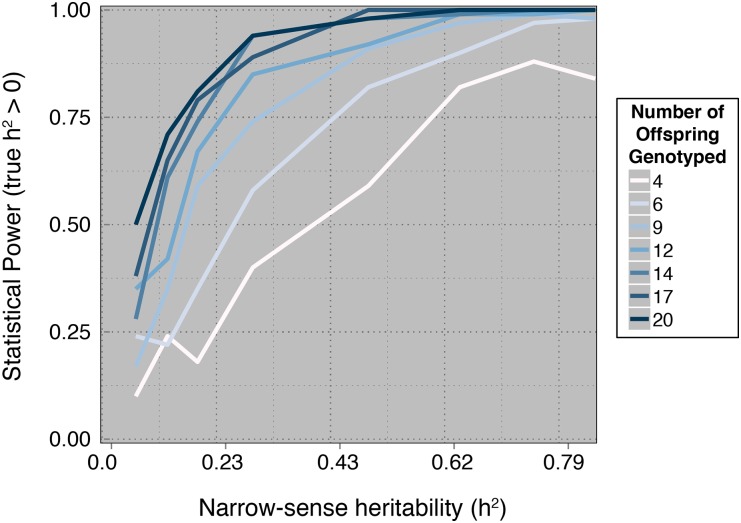
Power analysis for a varying number of offspring. The mean number of offspring genotyped per sire, *μ*, was varied between 4 and 20, whereas the size parameter for the negative binomial distribution was $\mu^{2}/\left(\sqrt{\mu \left(12.63/4.63\right)}-\mu \right)$. The number of sires was fixed at 9. The power to reject the null hypothesis of *h*^2^ = 0 is plotted as a function of narrow-sense heritability (*h*^2^), where the true value of *h*^2^ > 0.

Power analysis results suggest that increasing the number of sires is the most effective mechanism to increase statistical power. Unfortunately, for heritabilities less than 0.4, very large numbers of sires will be required. The intuition is that substantial amounts of variability between sires is expected just due to sampling alone, and therefore statistical support for a nonzero heritability requires large sample sizes. Despite the lack of statistical power, this approach does have the desirable property of low false-positive rates. For example, even with nine sires, we expect to have a nearly 90% chance of failing to reject the null hypothesis on data sets simulated with an among-sire variance equal to zero (Figure 4B). Lastly, if sequencing additional offspring is an option, statistical power can be improved (Figure 5).

## Discussion

In this paper, we present an experimental and statistical methodology for estimating the heritability of traits in nonmodel, highly fecund organisms. We applied this approach to determine whether settlement is a heritable trait in the reef-building coral *O. faveolata*. Although we did not find statistical support for a nonzero, heritability in this trait, a power analysis suggests we lacked a sufficient number of individuals. Our computational method includes code for fitting model parameters, performing model selection using a permutation test, and calculating the expected statistical power for proposed or completed studies. The power calculation method is especially important for studies requiring animal care and use approval and/or those with complex or expensive collection demands.

Previous work suggests that heritable variation exists for a variety of traits across many marine organisms (Foo *et al.* 2012; Johnson *et al.* 2010; Kelly *et al.* 2013; Lobon *et al.* 2011; McKenzie *et al.* 2011; Parsons 1997), including corals (Kenkel *et al.* 2011; Meyer *et al.* 2009). These studies have found significant heritability for nearly every trait measured in corals (Carlon *et al.* 2011; Kenkel *et al.* 2011; Meyer *et al.* 2009, 2011), but see Csaszar *et al.* (2010). In fact, one study specifically quantified the additive genetic variance in settlement rates of the Pacific reef-building coral *Acropora millepora* and found *h*^2^ = 0.49; however no variance around this mean was estimated (Meyer *et al.* 2009). It would not be surprising from an evolutionary standpoint if an ecologically important life-history trait such as larval settlement was heritable in other coral species, such as *O. faveoalta*. However, in this study we were unable to detect heritable variation, likely due to insufficient numbers of individuals.

There is a rich quantitative genetics literature on estimating the heritability of binomial traits dating back to Wright (1917) and Fisher (1918); however, the first use of generalized linear models fit to observed presence/absence data are from Gilmour *et al.* (1985), with key future contributions from Foulley *et al.* (1987) and Vazquez *et al.* (2009). These methods originally were developed for agricultural breeders, where fewer constraints exist on the number of families used to estimate the heritability, for example, the viability of poultry (Robertson and Lerner 1949), common genetic disorders of Holstein cows (Uribe *et al.* 1995), and root vigor in sugar beets (Biscarini *et al.* 2014, 2015). Uribe *et al.* (1995) estimated sire and residual variance components by using restricted maximum likelihood, or REML, modeling of 7416 paternal half-sib cows and found that heritability of common diseases in cows ranged from 0 to 0.28. These sorts of numbers are unreasonable to sample in natural populations of corals because parentage is rarely known unless controlled crosses are completed and then the costs associated with genotyping thousands of individuals are prohibitive.

A pair of recent papers by Biscarini *et al.* (2014, 2015) developed a cross-validation−based algorithm for selecting single-nucleotide polymorphisms that maximally classified sugar beets into high and low root vigor. Therefore, our principle contribution is in terms of model selection, in the form of a permutation test to determine whether statistical support exists for a nonzero narrow-sense heritability, and the methods application to nonmodel organisms. In such organisms, where breeding, collection, and/or budgetary constraints may exist, such a model-selection procedure is essential.

Our approach has three important caveats. First, as stated in *Materials and Methods*, one cannot disentangle additive variation due to sire from dam-specific sire effects under the sire model (see Lynch and Walsh (1998), for a detailed explanation). Therefore, conservatively, heritability estimates using our approach should be considered estimates of broad-sense heritability. Second, our methods are somewhat lacking in statistical power. For heritabilities thought to be typical of studies in nonmodel organisms, well more than 50 individuals may need to be typed across nine sires, see Figure 4A and Figure 5. However, our methods perform very well with respect to minimizing the type-I error rate, see Figure 4B. Lastly, as stated in the methods, the accepted approach—based on mixtures of χ^2^ distributions—has even less statistical power and was a poor approximation to our observed null distribution. Future work should focus on adapting existing methods and developing new methods to allow for smaller sample sizes. This effort is meant to be a project that will grow and develop organically; therefore, we welcome suggestions and contributions and plan regular updates to the statistical methods.
